# Transmigration of Mandibular Canine: Report of Four Cases and Review of Literature

**DOI:** 10.1155/2011/381382

**Published:** 2011-07-06

**Authors:** Gaurav Sharma, Archna Nagpal

**Affiliations:** ^1^Oral Medicine and Radiology Department, Sudha Rustagi College of Dental Science & Research, Faridabad, Haryana, 121002, India; ^2^Department of Oral Medicine and Radiology, PDM Dental College & Research Institute, Bahadurgarh, Haryana, 124507, India

## Abstract

Transmigration of canine is a rare phenomenon. The prevalence of transmigration of mandibular canine has been found to be only 0.14%–0.31%. The treatment of impacted transmigrated canine is very complicated if it is diagnosed at a later stage. We report 4 cases of transmigration of mandibular canine and review the literature regarding the etiology and treatment. Panoramic radiograph should be taken during the mixed dentition period if the mandibular canine has not erupted from more than one year from its normal chronological age of eruption as intraoral periapical radiograph examination will not always detect an impacted or transmigrated canine.

## 1. Introduction

Preeruptive migration of a tooth across the midline is termed as transmigration. The incidence of mandibular canines to migrate across the midline is rare. The prevalence of impacted maxillary canines has varied from 0.12% in a study conducted by Aras et al. [1] to 0.34% in another study [2]. The occurrence of transmigration of mandibular canines varies from 0.14% to 0.31% [2, 3]. According to Javid [4], an impacted mandibular canine that has crossed the midline more than half of its length should be considered as transmigrated. However, Joshi [5] stated that the tendency of a canine to cross the mandibular midline is a more important consideration than the distance of migration after crossing the midline. There are no clinical symptoms of transmigration, although follicular cyst formation and chronic infection with fistulization have been reported [4]. Due to transmigration aesthetics may be compromised, which might have psychological implications. Canines are considered as turning point in the dental arches. In this paper we have stressed upon the significance of screening panoramic radiographs to be taken during the mixed dentition period to evaluate the tendency of mandibular canines to transmigrate. We report 4 cases of impacted mandibular canines migrating through the midline and review the various aetiologies and management of transmigration and significance of early recognition.

**Case 1**

**Figure 1:**
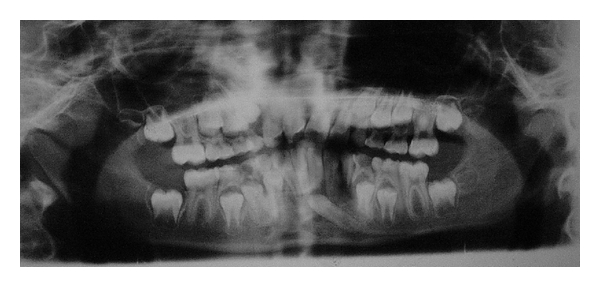
Panoramic radiograph showing tooth buds of all permanent teeth.

An 8-year-old female presented to department of oral medicine and radiology with a chief complaint of decayed teeth in lower right and left posterior region. A screening panoramic radiograph (Figure 1) revealed the tooth buds of all remaining permanent teeth in normal development positions according to her chronological age. The crown of 33 was observed to be crossing the midline. The patient's parents were informed regarding transmigrated canine and considering the young age at which transmigration was diagnosed, the immediate possibility of surgical extraction was not advised. The patient was referred for an orthodontic evaluation and the patient was subsequently recalled for periodic followup. The mandibular incisors were found to be fully erupted, and no evidence of any pathology was detected.

**Case 2**

**Figure 2:**
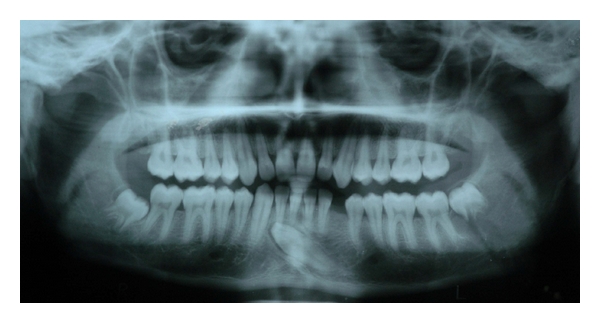
Panoramic radiograph showing transmigrated 33 lying below the apices of mandibular incisors.

A 17-year-old male reported to department of oral medicine and radiology with a history of trauma 3 days back. Panoramic radiograph (Figure 2) revealed an evidence of fracture extending from inferior border of mandible to developing tooth bud of 38. 33 was found to be crossing the midline and was below the apices of mandibular incisors with no evidence of any pathology or root resorption.

**Case 3**

**Figure 3:**
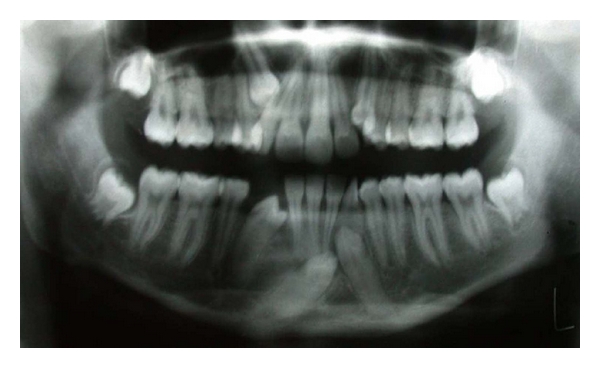
Panoramic radiograph showing 43 transmigrating towards left side.

An 18-year-old female complaining of missing upper and lower front teeth had come to department of oral medicine and radiology for orthodontic treatment. Panoramic radiograph revealed impacted 13, 33, 43, and a rotated 42 (Figure 3). 44 was found to be migrating mesially. Displaced 12 and 22 towards mesial direction were also observed. 43 was found to be crossing the midline and lying below the apices of 31 and 32. There was no evidence of any pathology or root resorption. Tooth buds of all the third molars could be observed in their respective positions. Patient was referred to department of orthodontics when needed.

**Case 4**

**Figure 4:**
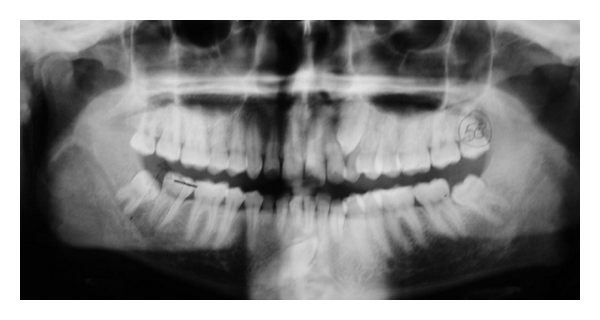
Panoramic radiograph revealing 43 transmigrating towards left side.

A 23-year-old male came to department of oral medicine and radiology complaining of missing teeth in maxillary and mandibular jaws. Panoramic radiograph (Figure 4) revealed overretained 63 and 83 with impacted 23, 43, 38, and 48. Displacement of 22 towards mesial side was also evident. 43 was crossing the midline and lying below apices of 31 and 32. There was no evidence of any pathology or root resorption. Patient was referred to department of orthodontics when needed.

## 2. Discussion

The permanent canines are the only teeth in which transmigration have been reported [6]. The larger cross-sectional area of the anterior mandible compared with anterior maxilla may be a reason for the higher frequency for mandibular transmigration [7]. Transmigration of maxillary canines is relatively uncommon perhaps due to the shorter distance between the roots of maxillary incisors and floor of nasal cavity. 

A specific aetiology of this anomaly is unknown, but traumatic factors, heredity, the long eruption path of canine tooth germs, premature loss of primary teeth, hypodontia, filling of this space by an adjacent tooth, disharmony of tooth size, unfavourable alveolar arch length, fractures with displacement of tooth bud, and long crowns can be the causative factors [6].

The term transmigration was first coined by Ando et al., and they also demonstrated the transmigration of a mandibular canine across the mandibular symphysis to the opposite side of the dental arch by serial radiographs taken over a period of several years [8]. The premature loss of teeth, inadequate arch space, and excessively large crowns were suggested as etiological factors [8]. If the angle formed by the midsagittal plane and unerupted canine is less than 30°, transmigration is unlikely. Those canines that lie between 30° and 50° may tend to cross the midline. When the angle exceeds 50°, crossing the midline becomes a rule [9]. Howard expected that the older patient would show a greater distance of travel because a longer time had been available for the migratory canine to travel [9].

Javid suggested that an abnormally strong eruption force, which drives the canine through the dense symphysis and that the conical shape of canine may be a cause of transmigration [4]. However, this hypothesis was rejected, as, by the time the canine migrates ectopically, the mandible has transformed into a single bone and the symphyses have been remodelled.

Vichi and Franchi suggested that agenesis of the adjacent teeth, in particular the lateral incisor, may favour retention of the primary canine and that the excess of space in the dental arch may account for the absence of a correct guide for eruption. They stated that the unerupted canine has the possibility of deviating from its normal developmental site, moving to a horizontal position, and migrating through the symphyseal bone only if enough space is available in front of the mandibular incisors [10].

 Odontomas are also suggested as a possible etiological factor [11]. Marks and Schroeder attributed initiation and control of eruption to the dental follicle at the molecular level, with the coronal portion stimulating bone resorption and the apical portion stimulating deposition. They suggested that a regional disturbance in the dental follicle may lead to local defective osteoclastic function with an abnormal eruption pathway being formed. This is a plausible explanation for aberrant eruption of teeth [6].

 Transmigrated teeth maintain their nerve connection to the originating side where the tooth germ is formed. Therefore, it is important to anesthetize the nerve on the originating side [12]. One case report described a patient who had severe pain during extraction of the transmigrated canine when the contralateral inferior alveolar nerve was not anesthetized [13].

So far, 196 cases of mandibular canine transmigration have been reported. Transmigration of tooth is generally a unilateral phenomenon, but 16 cases of bilateral transmigrations have been reported. There is a slight female predilection (1.6 : 1), and left side is involved more as compared to right side [14]. Mupparapu had used five criteria to classify the transmigrated canines [15]. These are summarized as follows.

**Type 1**

The canine is impacted mesioangulary across the midline, labial, or lingual to the anterior teeth with the crown portion of the tooth crossing the midline.

**Type 2**

The canine is horizontally impacted near the inferior border of the mandible below the apices of incisors.

**Type 3**

The canine has erupted either mesial or distal to the opposite canine.

**Type 4**

The canine is horizontally impacted near the inferior border of the mandible below the apices of either premolars or molars on the opposite side.

**Type 5**

The canine is positioned vertically in the midline with the long axis of the tooth crossing the midline.

Most of the cases reported in the literature are Type 1. All the cases in our report also exhibited Type I transmigratory pattern.

Various treatment modalities like surgical extraction of transmigrated canines, transplantation, exposure and orthodontic alignment have been suggested [6]. The most preferred treatment for migrated canines is surgical extraction. This is especially true when the mandibular arch is crowded and requires therapeutic extractions to correct the incisor crowding. If the mandibular incisors are in normal position with sufficient space for transmigrated canine, then transplantation can be undertaken [6]. Howard transplanted a transmigrated canine when there was enough space to accommodate the tooth [9]. 

 Wertz used orthodontic treatment to bring a labially impacted transmigrated canine into position [16]. However, if the crown of such a tooth migrates past the opposite incisor area or if the apex is seen to have migrated past the apex of the adjacent lateral incisor, it might be mechanically impossible to bring it into place. Abbott et al. suggested that the premature extraction of first premolars should be avoided when radiographs demonstrate the presence of an overlying mesially angulated unerupted canine that has begun to migrate labially across the incisors [17]. Taguchi et al. reported considerable improvement in the position of those canines associated with an odontoma, after removal of the odontoma and surgical exposure [10].

 Some authors, however, believe that symptomless, nonerupted teeth can be left in place [17]. In these patients, a series of successive radiographs should be taken periodically. A progressive worsening of the position of the unerupted canine or suggestion of cystic change of the follicle should consider the possibility of surgical extraction. The existence of pressure resorption of the roots of adjacent teeth, periodontal disturbances, or other possible foci for the spread of infection, prosthetic problems, malposition of the adjacent teeth, and neuralgic symptoms have been included as indications for surgical intervention in cases of impacted mandibular canines [6].

There were an equal number of males and females affected (2 males and 2 females), and no side preference could be observed (2 right side and 2 left side). This was in accordance with a study conducted by Aktan et al. in 2010 [2]. However in a recent study conducted in Greek population a slight left-side predilection was observed [18]. The average age of our reported cases was found to be 16.5 years. This figure was comparatively lower as compared to average age of 29.6 years in a study conducted by Aktan et al. in 2010 [2].

 In the four cases presented in this report, two cases exhibited other dental abnormalities (Table 1). The anomalies reported in patient 3 were impacted maxillary and mandibular canines along with mesially aligned mandibular premolar on right side. In Case 4 maxillary impacted canine was observed along with overretained deciduous teeth. There was no associated pathology with transmigrated canines as well as impacted canines reported in patient 3 and patient 4. Presence of retained deciduous teeth in patients 3 and 4 maybe suggested as a cause of transmigration. However, the exact cause of transmigration cannot be ascertained because of no previous clinical and radiographic records. 

Patients presenting with transmigration have an age range of 8–62 years [6, 12]. This observation shows that migration typically starts at age of 6–8 years when root formation has not occurred completely. The presence of transmigration must be suspected, if the permanent mandibular canine is absent from the arch or is more than one year behind the normal eruption schedule. Intraoral periapical radiographs will not always reveal an impacted canine or transmigration. Therefore the importance of panoramic radiograph as a screening radiograph, when there is delay in eruption of permanent canine for more than one year, cannot be ignored when taking the proper radiation protection measures, as at later stage the treatment of transmigrated tooth will be more complicated. This would outweigh the radiation risk if impacted or transmigrated canine is found, and we consider the complications associated with transmigration and its complicated treatment.

In a screening panoramic radiograph if taken for routine purpose, it is advisable to always check for angulation of mandibular canine with midsagittal plane. If the angulation is above approximately 30°, patient should be recalled for periodic evaluation after every 3 months. In Case 1 patient was kept for periodic followup. Reimplantation of transmigrated canine was planned in Case 2 but the patient was not willing for any kind of treatment. In Case 3 and 4 removal of retained deciduous teeth was planned, and surgical intervention was advised.

## 3. Conclusion

Transmigration of the mandibular canine is a rare event, and early radiographic examination of a patient is important for treatment planning. An intraoral periapical radiograph may not be sufficient to detect transmigration and should be supplemented by a panoramic radiograph, especially in a mixed dentition stage if the permanent canine has not erupted for more than one year. Local anaesthetic must be administered on the side from which the transmigrated tooth originated before surgical extraction. Angulation of long axis of unerupted canine with midsagittal plane should always be evaluated in mixed dentition period. An early and timely intervention would lead to better management of transmigrated canine and hence avoids the potential complications associated with transmigrated canine.

## Figures and Tables

**Table 1:** Brief summary of the 4 transmigrated cases.

Case no.	Age (in years)	Gender	Transmigrated canine	Coexisting dental anomalies
1	8	Female	33 (Unilateral)	—
2	17	Male	33 (Unilateral)	—
3	18	Female	43 (Unilateral)	Impacted 13, 33, 43 and rotated 42, Mesially migrating 44
4	23	Male	43 (Unilateral)	Overretained 63 and 83, impacted 23, 43, 38, 48
